# Cardiac Amyloidosis Responding to Bortezomib: Case Report and Review of Literature

**DOI:** 10.2174/157340309788970360

**Published:** 2009-08

**Authors:** Edriss Charaf, Said B Iskandar, Ashley Blevins, Bernard Abi-Saleh, Stephen Fahrig

**Affiliations:** 1 Division of Cardiology, University of South Alabama, Mobile, Alabama, USA; 2 Halifax Heart Center, South Boston, VA, USA; 3 Department of Anesthesiology, Wake-forest University, Winston-Salem, NC, USA; 4 St Thomas Hospital, Nasheville, TN, USA

## Abstract

We report a case of a 53-year old patient with symptoms of congestive heart failure in whom a restrictive cardiomyopathy and a kappa-chain monoclonal gammopahty were diagnosed. Treatment with eight cycles of Bortezomib, a proteasome inhibitor, resulted in a significant regression of myocardial amyloid deposition and a notable clinical and hemodynamic improvement. Over the last few years, the management of cardiac amyloidosis has taken advantage of many of the advances of the chemotherapeutic regimens, as well as the wider availability of stem cell transplantation. The management of cardiac amyloidosis is also expected to evolve and improve with the better understanding of the specific mechanisms of amyloidogenesis and myocardial deposition. This will probably make certain molecules targeting specific sites in this process, as potentially effective and minimally toxic compared therapy with the currently used ones. In this article, we describe one of the first reported cases of cardiac amyloidosis, successfully treated with Bortezomib. We describe and discuss the mechanisms of action of Bortezomib and provide a detailed review of cardiac amyloidosis, from pathophysiology to diagnosis and treatment.

## CASE PRESENTATION

**Fig. (1):**
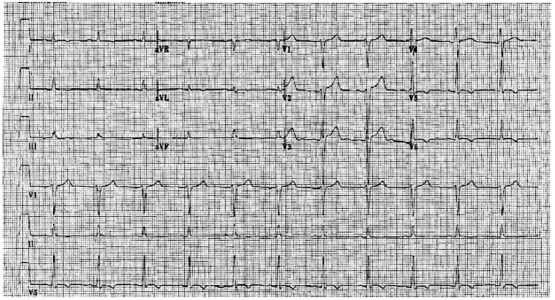
Electrocardiogram on the day of admission showing low voltage in the limb leads.

We describe the case of a 53-year old male patient with no known cardiac risk factors, who presented to our emergency room for a new onset of dyspnea on exertion and chest discomfort. His past medical history was unremarkable except for a history of bilateral carpal tunnel syndrome, for which he underwent bilateral median nerve decompression 4 years before this presentation. At that time, he underwent a simple median nerve relief surgery with no pathological examination. His family history was unremarkable; he denied tobacco, alcohol or over-the-counter medications use. Clinical examination was significant for crackles over lung bases, III/VI systolic ejection murmur over the apex with no pericardial knock or rubs heard. There was no palpable organomegaly but grade I-II lower extremity edema. His neurological examination was unremarkable. His cardiac enzymes were negative but BNP was elevated at 1270 pg/ml. His chest X-Ray revealed cardiomegaly and mild bilateral pulmonary congestion. A 12-leads electrocardiogram (Fig. **1**) revealed low voltage in the limb leads with non-specific inferior and lateral ST-T changes.

**Fig. (2):**
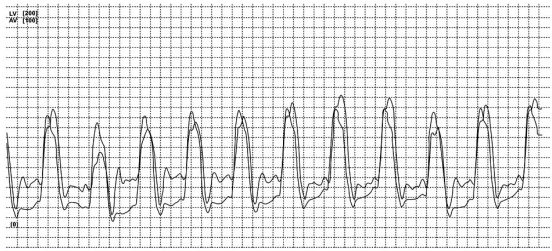
Right and left ventricular tracing with concordant change in pressures during expiration and inspiration confirming the diagnosis of restrictive cardiomyopathy.

A transthoracic echocardiography showed a thickened interventricular septum and posterior wall at 1.9 cm, high normal internal ventricular diastolic and systolic dimensions at 5.6 cm and 4.0 cm respectively and moderately enlarged left atrium at 5.1 cm. Two-dimensional imaging revealed also an increased left ventricular size and mass with a “granular sparkling” myocardial appearance. The Left ventricle was globally and moderately hypo-kinetic with an estimated ejection fraction of 35%. Doppler examination revealed moderate mitral regurgitation. Mitral inflow pattern was consistent with restrictive physiology with lack of respiratory variation. The right ventricular systolic pressure was 40 mmHg. A restrictive cardiomyopahty secondary to a systemic amyloidosis was suspected. A left and right heart catheterization consistently revealed a mild global hypokinesis with an estimated ejection fraction of 40% and a 2 to 3+ grade mitral regurgitation. Right and left ventricles hemodynamic evaluation (Fig. **2**) revealed a concordant increase and decrease of both pressures with respiration, suggestive of a restrictive physiology. It also showed elevated right chambers pressures with right atrial pressure 20mmHg, right ventricular pressure of 56/20 mmHg, pulmonary capillary wedge pressure (PCWP) of 33 mmHg and mean pulmonary arterial pressure (MPAP) of 40 mmHg.

**Fig. (3):**
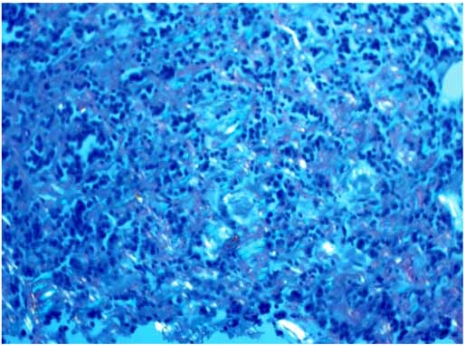
Congo-Red stain done on the patient’s bone marrow biopsy, showing focal apple-green fluorescence under polarized light, consistent with amyloid deposits.

A bone marrow biopsy showed a medullary plasmacytosis (10-15%) and stained positive for amyloid with Congo-red staining (Fig. **3**). Serum immunofixation electrophoresis showed a discrete monoclonal IgG Kappa band and a serum kappa-chain concentration of 2,226. Urine protein electrophoresis (823 mg of protein collected in 24 h) showed a discrete band of Kappa light chain. Myocardial biopsy was deferred by the patient. A skeletal survey showed no lytic lesions. The diagnosis of kappa-chain systemic amyloidosis with cardiac involvement was made based on the simultaneous presence of the following findings: the presence of monoclonal kappa-chain producing bone marrow plasma cells, the electrocardiographic and echocardiographic proof of myocardial infiltrative disorder highly suggestive of amyloidosis, and the restrictive hemodynamic findings on right and left cardiac catheterization. The combination of these findings with our patient clinical presentation provided a quite convincing explanation for the etiology of his restrictive cardiomyopathy, precluding the need for an endomyocardial biopsy.

**Fig. (4):**
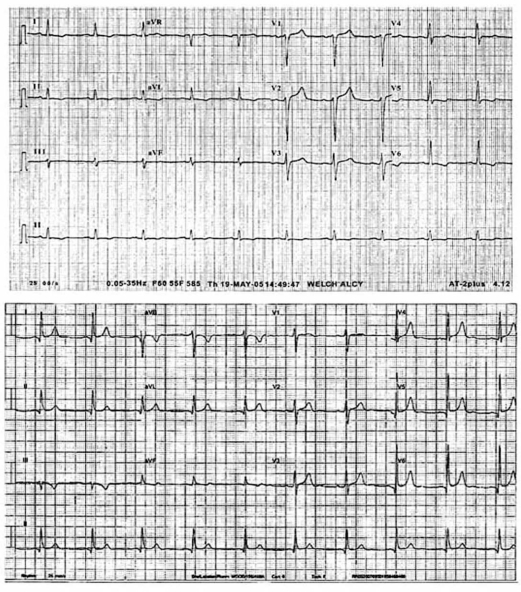
ECGs at14 and 24-month of follow-up, showing progressive resolution of microvoltage previously seen on limb leads.

The patient was deemed ineligible for stem cell transplantation. Chemotherapy targeting kappa-chain amyloidosis was initiated. Eight cycles of Melphalan and high dose of Dexamethasone were administered. After several months of therapy, this regimen has failed to improve our patient’s performance status, ejection fraction (35%) or reduce ventricular wall thickness. A trial of Thalidomide was initiated but ended quickly because of significant intolerance and the development of deep venous thrombosis. Bortezomib was then started at a dose of 1.3 mg/ m2 for eight cycles over 5 months. Like other cytotoxic agents traditionally used in the treatment of AL amyloidosis, bortezomib is know to target neoplastic cells and enhance their apoptosis through different mechanisms as detailed later in our review. The end result will be a reduction of the production and burden of amyloidogenic proteins, minimizing their deposition in various tissues including the myocardium. This regimen was well tolerated and no major side effects were observed. After 12 weeks of treatment, the patient reported a significant improvement in his functional capacity, exercise tolerance, and significant improvement of his exertional dyspnea and chest discomfort. The clinical improvement correlated with the disappearance of monoclonal spikes on serum and urine electrophoresis with a significant reduction in serum Kappa and Lambda light chains concentration. His follow-up electrocardiogram is shown below (Fig. **4**). A follow-up echocardiography showed a significant regression of amyloid myocardial infiltration, decreased interventricular septum and posterior wall thickness from 1.9 to 1.3 cm, decreased left atrial diameter from 5.1 to 4.7 cm, and improvement of left ventricular ejection fraction from 35% to 55%. Until last seen in his scheduled appointment, the patient remained in good status and will continue to follow at our facility.

## CARDIAC AMYLOIDOSIS

### Introduction

Amyloidosis refers to heterogeneous diseases, caused by protein conformation disorders in which different soluble proteins aggregate as extracellular insoluble fibrils in different tissues and organs [1]. The disease’s symptoms and manifestations are broad and insidious, delaying the diagnosis and often causing significant organ dysfunction before a diagnosis is made. Organ dysfunction results from both disruption of tissue architecture by amyloid deposits and direct cytotoxic effects of light chains [2]. The prognosis is mainly determined by the presence and extent of heart involvement as well as the response to therapy [3]. Cardiac involvement is a common finding and is the most frequent cause of death in Amyloidosis [4]. The incidence of cardiac involvement is 50 % in patients with AL (Immunoglobulin light chain disease) Amyloidosis. It is however less frequent in AA Amyloidosis (serum Amyloid A protein deposition disease) and the familial syndromes with 10% and 5% respectively. In these setting, it is usually associated with milder disease [5]. It accounts for 10% of causes of non-ischemic cardiomyopathy with significantly high mortality rate of up to 50 % [6]. Clinically apparent heart disease is present in one third of patients although the heart is virtually always involved when studied pathologically [4]. It is estimated that 25 % of the myocardial mass is replaced with amyloid at the time the patient exhibits symptoms and signs of heart failure [6].

### Classification and Diagnosis

Acquired systemic amyloidosis occurs in more than 10 per million person-years in the U.S. population [7] and is probably much under diagnosed in the elderly population. Systemic AL amyloidosis is the most serious and commonly diagnosed form, with 2,000 to 2,500 newly diagnosed cases annually in the United States [8]. The classification of cardiac amyloidosis is based on the nature of precursor protein.

1. AL Amyloidosis (Immunoglobulin light chain disease): the most common and aggressive form of amyloidosis. Its incidence is around 8.9 per million, with peak onset around the sixth decade and male to female ratio of 3:2 [9, 10]. Theoretically, any organ can be infiltrated by light chain. The kidney and the heart remain the most common organ involved whereas other organs like liver and peripheral and autonomic nervous system are much less commonly involved. It is estimated that at least two-third of patients with AL amyloidosis have more than one affected organ when the diagnosis is made [11] and less than 5% of patients have clinically isolated heart involvement [13]. The light chains are formed by part or the whole variable (VL) domain of the immunoglobulin [14]. The λ/κ chain ratio higher than 2:1 in AL amyloidosis and may reach 4:1 in cases of cardiac involvement [15]. Due to conformational changes, monoclonal light chains aggregates into amyloid fibrils, leading to their deposition in any organ, except brain tissue [11, 16]. The conformational change is the result of a change in the amino acid sequence or a proteolysis event, making the light chain vulnerable to thermodynamic changes leading to self aggregation [2]. Light chain secretion and organ infiltration are associated in 80% of cases with a benign monoclonal gammopathy [14] but could also be secreted by multiple myeloma, lymphomas, and macroglobulinaemia. Cardiac involvement is found in 60 to 90% of patients with AL amyloidosis, up to half of cases presenting with advanced symptoms of right sided heart failure and echocardio-graphic evidence of diastolic dysfunction, reflecting the aggressive nature of the disease [9, 14]. The estimated median survival is as low as 4 months with most deaths being due to refractory congestive heart and arrhythmias [9].
2. Systemic AA or secondary amyloidosis is caused by the deposition of reactive Amyloid “A” fibrils, produced from an underlying inflammatory or neoplastic process. While cardiac involvement is usually rare and mild, the kidney involvement manifested by proteinuria and renal failure is very common [14]. The associated organ dysfunction is likely to resolve after the treatment of underlying disease [9] and usually reflected by a reduction in the SAA concentration and results in improved survival [16].
3. Hereditary amyloidosis is caused the deposition of a mutant transthyretin protein synthesized by the liver. Other less common amyloidogenic proteins include apolipoprotein A-I, lysozyme, fibrinogen alpha-chain and other rare proteins [14]. An Isoleucine 122 gene mutation of TTR DNA is known to be associated frequently with a milder degree of cardiac involvement compared with AL amyloidosis [9] and is present in 4% of African-Americans, and 23% of African-Americans with cardiac amyloidosis have this variant [16]. Other manifestations include peripheral neuropathy and nephropathy.
4. Senile systemic amyloidosis with cardiac involvement is seen in 25% of persons older than 80 years. It is caused by the deposition of normal transthyretin in multiple organs a mainly atrial cardiac tissue [9, 10]. It is usually clinically unrecognized until massive tissue deposition occurs and leads to heart failure. However, an estimated median survival at 75 month reflects its milder course and favorable prognosis [9].
5. Hemodialysis-associated amyloidosis occurs with chronic hemodialysis and is characterized by the deposits of the Beta-2 microglobulin amyloid fibril subunit mainly in skeleton and joints and minimal myocardial involvement.
6. Isolated atrial amyloidosis without systemic involvement is a common finding at autopsy defined as a thin endocardial layer. It is commonly seen in elderly patients with chronic atrial fibrillation and valvular disease and its clinical significance is unknown.

The differentiation between these subtypes done based on immunohistochemical and genetic testing helps to tailor the therapeutic strategy and defines the prognosis.

### Pathophysiology and Clinical Manifestations

Cardiac amyloid infiltration affects contractility, electric conduction and coronary flow. On autopsy, the most common finding is mild atrial enlargement usually without significant ventricular dilatation. Thickened myocardium, enlarged chambers, intra-cardiac thrombi, valvular leaflets and coronary vessels infiltration are other less common findings. The extensive deposition in atrial walls results in mechanical failure and mechanical standstill [9]. The most common manifestation of myocardial dysfunction in cardiac amyloidosis is restrictive cardiomyopathy and diastolic dysfunction. This is mainly due to the direct effect of amyloid deposition on myocardial contraction and relaxation; a light chain-induced oxidant stress may be also involved as well [5]. Systolic dysfunction is the second most common presentation, usually when more than 25 % of the myocardial mass is replaced by amyloid deposits. Atrial dysfunction is mainly related to the impairment of atrial emptying due to loss of systolic function and increased after load, even in the presence of sinus electric activity. The most severe condition is defined as atrial standstill or atrial electromechanical dissociation [5]. Sudden death, accounts for 30-50 % of all cardiac deaths in systemic amyloidosis, usually secondary to ventricular arrhythmias, atrio-ventricular block and acute electromechanical dissociation. Survival to discharge is low because of recurrent ventricular arrhythmias [18]. Prolonged HV interval and late potentials may independently predict the risk of sudden cardiac death [5]. The data about presence and character of vascular abnormalities in cardiac amyloidosis is still limited. Amyloid infiltration of small intramural vessels is associated with endothelial dysfunction and may explain the anginal chest pain in patients with normal coronaries [19]. Modesto *et al.*, found in a recent prospective trail involving 59 patients with cardiac amyloidosis, an increased carotid artery intimal-medial thickness (IMT) and lower brachial artery flow-mediated dilatation (FMD) with an incidence of 88-90% of AL patients. However, this finding did not correlate with the echocardiographic findings of cardiac amyloidosis [19].

### Clinical Manifestations

The restrictive physiology in cardiac amyloidosis results in rapid and progressive symptoms and signs including mainly progressive dyspnea and chest discomfort. Angina due to amyloid deposits in the small and intramyocardial coronary arteries is not uncommon. Signs of right sided heart failure predominate, with peripheral edema, elevated jugular venous pressure with prominent X and Y descent, mitral and tricuspid regurgitation murmurs, narrow pulse pressure in the setting of restrictive cardiomyopathy. S4 is uncommon and is most likely attenuated because of the reduced systolic function of the atrial myocardium caused by amyloid infiltration. Syncope may be the presenting complaint and is usually multifactorial caused by or orthostatism, brady- or tachyarrhythmia. Orthostatism results from a decreased ventricular filling caused by the underlying restrictive pathophysiology. It is not uncommon for pre-existent hypertension to resolve spontaneously as the amyloidosis progresses [4, 5]. Sudden death, a common presentation in patients with advanced cardiac amyloidosis, may be caused by electromechanical dissociation rather than ventricular arrhythmia [5, 4]. 

### Diagnosis

The diagnosis of cardiac amyloidosis requires an evidence of amyloid tissue involvement, usually in abdominal fat, rectal mucosa or bone marrow, plus the demonstration of an underlying plasma cell clone. Despite the strong evidence provided by the finding of amyloid deposits in a biopsy in the presence of monoclonal immunoglobulin, it is essential to exclude any underlying non-AL amyloidosis associated monoclonal gammopahty [11].

#### Electrocardiography and Electrophysiologic Studies

Pseudoinfarction and low voltage on ECG are the most frequent electrocardiographic findings, present in about 47% and 46% respectively in patients with biopsy-proven cardiac amyloidosis and do not seem to reflect a low ejection fraction [14]. Pseudo-infarction pattern with small or absent R waves in right precordial leads and less frequently Q waves in the inferior leads are not very specific for cardiac amyloidosis and occur in the setting of emphysema, hypothyroidism, hypoadrenalism, pericardial effusion and obesity. The cardiac conduction system is frequently affected by amyloid infiltration. His-Purkinje system is the most frequently affected parts. Right bundle branch block occurs more frequently than left due to its greater susceptibility to be affected by the amyloid infiltrates, making it uncommon to have an isolated left bundle branch block [4, 20]. H-V prolongation > 55 ms may be missed in the setting of a narrow QRS. Signal-averaged electrocardiogram may reveal delay myocardial activation by showing late potentials whose incidence correlates with the echocardiographic evidence of cardiac amyloidosis (31% correlation rate versus 9 % of patients with normal echocardiogram) and the risk of sudden cardiac death [4]. Another prognostic electrocardiographic marker may be the presence of reduced heart rate variability on 24-h Holter electrocardiogram, being associated with an increased short-term mortality [21]. Atrial fibrillation and flutter are the most common arrhythmias encountered and happen more frequently in patients with echocardiographic evidence of increased left ventricular wall thickness [14].

#### Cardiac Biomarkers

Brain Natriuretic Peptide (BNP) expression was found to be increased in myocytes adjacent to amyloid deposits. This is explained by regional stress effect of amyloid infiltration on myocytes and increased filling pressures [22, 23]. In cardiac amyloidosis, serum BNP levels are usually high, but out of proportion of the degree of heart failure symptoms. Serum BNP levels may correlate well with the degree of myocardial amyloidal infiltration, but it’s unknown if it correlates with the disease’s evolution. It was found that even a sub-clinical cardiac amyloid infiltration may produce elevated BNP level > 100 pg /ml in 21% of patients without echocardiographic evidence of cardiac amyloidosis [22]. BNP and N-terminal pro-BNP concentrations are closely linked. N-pro-BNP concentration of more than 152 pg/ml detected cardiac involvement with a sensitivity and specificity of 93% and 90% respectively. Patients with N-Pro-BNP levels higher than 152 pg/ml also have a higher all cause mortality rate (72, 2 % versus 7.6% per year in those whose N-Pro-BNP levels were lower than 152 pg/ml) [5]. Elevated serum troponin can also be elevated in cardiac amyloidosis, due to endothelial dysfunction, microinfarctions and small vessels intramural obstruction. Troponin T/ I and N-terminal-ProBNP levels are of prognostic value in patients with amyloidosis and a detection of cardiac troponin I or T conferred a median survival of 6 or 8 months (respectively), compared with 22 or 21 months, respectively, in those without any detectable troponin I/T [24]. Despite the finding that NT-proBNP values may decrease rapidly following effective chemotherapy and were associated with improved event-free survival [25], their usefulness in monitoring the disease progression is not well established yet [9]. Another promising newer marker, serum immunoglobulin free light chains assay, recently investigated in patients with AL amyloidosis, showed a sensitivity which falls between 85 and 98% [25] and may be used to serially monitor production of amyloidogenic light chains during chemotherapy [14]. The benefit of using serum markers will be probably the greatest in following response to chemotherapy. This may require coupling these tools with other reproducible quantitative imaging modality like CMR. 

#### Echocardiographic Findings

Echocardiography is an important tool in the diagnosis with a broad spectrum of sonographic and hemodynamic findings. While it is not diagnostic alone, the specificity of the echocardiographic findings increases in the presence of the clinical manifestations suggestive of myocardial amyloidosis. Diastolic dysfunction, suggested by reduced diastolic mitral inflow velocities upon Tissue Doppler imaging, is the earliest finding in cardiac amyloidosis [26, 27, 14]. The findings of increased thickness of ventricular walls along with a granular sparkling texture of the myocardium are strongly suggestive of cardiac amyloid infiltration with a specificity approaching 81% [14]. However, this finding is present in only about fourth of the patients with amyloid cardiac involvement and can be found in other conditions like hypertrophic and hypertensive cardiomyopathies. The addition of the finding of increased interatrial septal diameter remarkably increases the specificity of ventricular wall thickness parameter [6, 14]. The thickness of the left ventricular walls correlates with reduced survival [9]. Similarly, an interventricular septum thickness of more than 15 mm is also considered a poor prognostic sign [5]. The association of increased ventricular thickness with low voltage on the electrocardiogram (Low voltage/mass ratio) strongly favors the diagnosis of amyloid myocardial infiltration [6, 4]. The sensitivity of an index combining a low voltage with interventricular septum thickness more than 1.98 cm, detects amyloidosis with a sensitivity and specifity of 72% and 91% respectively [5]. A combined typical clinical presentation of cardiac amyloidosis with specific echocardiographic findings such as dilated atria, interatrial septal hypertrophy > 7 mm, thickened valves and right ventricular free wall may be diagnostic of cardiac amyloidosis even without an endomyocardial biopsy [5]. In addition to its important role in early detection of diastolic dysfunction, Tissue Doppler imaging is very helpful distinguishing amyloid or any restrictive cardiomyopathy from constrictive pericarditis. The finding of a mitral annular diastolic velocity (E’) less than 8 cm/s is suggestive of restrictive rather than constrictive physiology [14, 28]. This finding may be the result of mitral valve amyloid infiltration causing intrinsic atrial dysfunction which is reflected by a diminutive A wave with a normal deceleration time [20]. Newer echocardiographic parameters are being developed to facilitate an early detection of ventricular dysfunction. Tei index is obtained from the tricuspid and pulmonary Doppler flow velocity and is defined as ICT + IRT/ ET (ICT = Isovo-lumic Contraction Time, IRT = Isovolumic Relaxation Time, and ET= Ejection Time). Tei index is usually increased in the cardiac amyloidosis and has been shown to be an independent prognostic marker [6, 28]. Tissue Doppler Imaging (TDI), strain rate imaging and ultrasonic tissue characterization are useful in demonstrating the evidence of longitudinal systolic dysfunction earlier than any decrease in the left ventricular ejection fraction [20].

#### Cardiac Catheterization

The non-invasive cardiac investigations are usually sufficient for the diagnosis of cardiac amyloidosis. The usual indication of cardiac catheterization is endomyocardial biopsy and hemodynamic assessment that may provide important clues for early diagnosis. According to the ACC/AHA guidelines [32] and may be of a prognostic value by defining the extent of tissue infiltration [4]. Both constrictive pericarditis and restrictive cardiomyopathy may have similar findings of early rapid diastolic filling and elevation with end equalization of diastolic pressures in all four cardiac chambers. However, the respiratory changes in filling of the left ventricle and right ventricle, as well as the enhanced ventricular interaction seen in constrictive pericarditis, result in distinctive changes in the left ventricular and right ventricular pressures during respiration. These abnormalities have been described as discordance (in patients with constrictive pericarditis) or concordance (in patients with restrictive cardiomyopathy) between the pressures in these two chambers, typically seen as reciprocal changes in stroke volume, pulse pressure, or peak systolic pressure during respiration. The coronary angiogram is usually normal because the low incidence of epicardial vessels involvement.

#### Myocardial Scintigraphy

Scintigraphy with technetium-99m pyrophosphate and other agents that bind to calcium is often strongly positive with prominent amyloid involvement. Although it lacks for consistent sensitivity, a positive scans correlate with significant cardiac involvement and is not part of the routine workup for cardiac amyloidosis. Technetium Tc 99m-3,3-Diphosphono-1,2-propanodicarboxylic acid scan was recently suggested to be capable of differentiating TTR-associated amyloidosis from AL amyloidosis [9].

#### Cardiovascular Magnetic Resonnance in Cardiac Amyloidosis

Cardiac Magnetic Resonance is a promising non-invasive diagnostic tool for cardiac amyloidosis. Its biggest advantage so far, is the ability to differentiate amyloidosis from other forms of restrictive cardiomyopathy like sarcoidosis and lymphoma-related infiltrativedisease or from hypertrophic cardiomyopathy. The most characteristic pattern, found in approximately two-thirds of patients, is the subendocardial late enhancement. This finding may be due to tropism of amyloid fibrils for the subendocardium (42%) and less frequently to the midwall and subepicardium, with 29% and 18%, respectively [28]. This technique allows not only “mapping” the transmural histological distribution of amyloid protein but also estimating the cardiac amyloid load. A higher amyloid load and therefore a shorterlongitudinal relaxation time correlate with some echocardiographic parameters such as LV mass, wall thickness, interatrial septal thickness, and diastolic dysfunction [14]. It is expected that cardiac magnetic resonnance (CMR) to be standardized for future use in screening of early sublinical amyloid infiltration and treatment response monitoring.

### Management

The optimal treatment of immunoglobulin light chain amyloidosis (AL) patients requires an (1) early diagnosis, (2) an accurate amyloid phenotyping, (3) an effective treatment aiming optimally to abolish the source of light chains, induce a regression of amyloid deposits and prevent further organ damage, and finally (4) maintenance with a supportive medical therapy [11, 14]. Since the management and prognosis depends upon types of amyloid fibrils, an accurate definition of fibrils type is a critical part of care.

#### I Medical Management

The management of heart failure in patients with amyloidosis is challenging since it involves the treatment of symptoms resulting from a restricitive cardiomyopahty. Judicious diuretics use and salt restriction with avoidance of intravascular volume depletion remains the mainstay of the treatment. High dose are required in the setting of the frequently associated nephrotic syndrome. Angiotensin-converting enzyme inhibitors and Angiotensin-II receptor blocker are poorly tolerated in cardiac amyloidosis associated congestive heart failure. This is due to the important role of the Angiotensin-II in maintaining the blood pressure and the frequently associated autonomic dysfunction precipitating orthostatic hypotension if not carefully introduced. The role of Calcium channel blockers and digoxin is limited and probably detrimental. This is due to an exaggerated negative inotropic effect with increased the avidity of amyloid fibrils for both molecules. (Just split the sentence). A high incidence of sudden death in patients treated with digoxin has been reported [6, 20]. A concomitant increase in both thromboembolic and bleeding risk is present in patients with cardiac amyloidosis [14]. An increased bleeding tendency is usually caused by vascular and capillary fragility due amyloid infiltration and other associated coagulation abnormalities [6]. On the other hand, an underlying hypercoagulable state along with an associated dilated cardiomyopathy increase the thromboembolic risk. Besides atrial fibrillation and the evidence of intracardiac thrombi, the presence of dysfunctional atrial contractions or a markedly dilated atrium caused by extensive amyloid infiltration may also be an indication for anticoagulation. This may be demonstrated on echocardiography (transthoracic and transesophageal) by the presence of a small transmitral A wave (< 20 cm/s) with a markedly decreased atrial appendage Doppler velocities (< 40 cm/s) [20]. Patients with symptomatic bradyarrhythmias should receive a permanent pacemaker implantation. In contrast to AL amyloidosis, familial and senile amyloidosis is associated with a higher incidence of conduction disturbances. However, the atrial and ventricular pacing threshold may be higher than usual in some patients. Treatment of ventricular arrhythmia in cardiac amyloidosis is not standardized, and the safety of antiarrhythmic agents is not well known. The use of devices like pacemakers and ICD in cardiac amyloidosis is still not well investigated. Deciding to implant an automated implantable cardioverter-defibrillator is difficult in these patients due to their poor prognosis. The available limited clinical experience suggests that pacemaker or implantable cardioverter-defibrillator (ICD) implantation often fails to prevent sudden cardiac death. This is due to the high incidence of electro-mechanical dissociation as a common cause of sudden cardiac death in this patient’s population [12].

#### II Specific Treatments

##### Chemotherapy

Chemotherapy aims to rapidly reduce or abolish the supply of the amyloidogenic monoclonal light chain from the underlying plasma cell clone, facilitate the regression of amyloid deposits and sustain the function of the organs involved. This often leads to improved quality of life and survival mainly in patients in whom at least 50% reduction in monoclonal light chain concentration is achieved [11, 29, 30]. Cardiac amyloidosis management took advantage of the recent validation of circulating free light chain concentration (FLC) as a marker of response to chemotherapy [11]. A consensus panel from the International Society for Amyloidosis established the criteria for hematological response [11] with complete response being defined by a 1) Negative serum and urine for monoclonal protein, 2) Normal free light chain ratio, and 3) Bone marrow plasma cells < 5%. A partial response is defined by the persistence of serum monoclonal component > 0.5 g/dl, light chain in the urine with a visible peak and >100 mg/day, and free light chain >100 mg/L (At least 50% reduction in each parameter). Other experts suggest that survival may be better predicted using the absolute value of FLC achieved after autologous stem cell transplantation (ASCT) rather than the percent reduction of FLC [11]. A greater drop in NT-pro-BNP level with complete remission also seems to correlate with FLC [11]. For AL Amyloidosis, the highest success rate occurs with melphalan with a complete hematological remission in 40 % of treated patients after one year follow-up [20]. In patients with advanced cardiomyopathy, the overall poor prognosis and the benefit of the chemotherapy should be balanced with a pretreatment mortality risk of more than 30%. An ejection fraction of less than 40% is considered a predictor of poor outcome and is considered a relative contraindication for chemotherapy. Other markers include a markedly elevated serum BNP, persistently elevated troponin, and evidence of pleural effusion or a significant myocardial thickening [20]. The other obstacles encountered in less severe cases consist of the poor tolerance of the chemotherapeutic agent or the presence of resistant clone [14]. In patients with a predicted poor outcome, the use of lower dose of Melphalan-based regimens is recommended and better tolerated but response is usually delayed [17]. The combination of oral Melphalan and high dose Dexamethasone showed remission in 33% of patients ineligible for stem cell transplantation [10]. Alternative regimens for Melphalan-refractory cases, is the combination of Thalidomide and Dexamethasone [10]. Other drugs under investigations include 4’-iodo-4’ Deoxydoxorubicin and Interferon- alpha remain under investigation. 

The treatment of secondary amyloidosis with cardiac involvement starts with the treatment of the underlying inflammatory or neoplastic process and usually carries a poor prognosis. The pharmacological therapy for familial amyloidosis is limited, given the production of abnormal protein, Transthyretin, by the liver. So, liver transplantation remains the most effective therapeutic option. Non-Steroidal Anti-Inflammatory Drugs are thought to be effective in vitro, stabilizing the native protein and preventing the deposition of transthyretin amyloid fibrils [10]. Senile cardiac amyloidosis treatment is similar to that of congestive heart failure, and prognosis is usually favorable.

##### Solid Organ Transplantation

Heart transplantation remains a controversial option because of the systemic involvement and the potential recurrence of graft amyloidosis [9, 10]. 

In primary amyloidosis, heart transplantation is only a palliative procedure and consequent supportive chemotherapy should be considered [10]. Long-term prognosis is poor (39% survival at 4 years in one study and 30% at 5 years in another, even with adjuvant chemotherapy [4, 9, 10]. Sequential heart and autologous stem cell transplantation for primary amyloidosis has been reported [10]. Liver transplantation remains the most effective therapy for hereditary amyloidosis, by removing mutant TTR proteins.

##### Stem Cell Transplantation

Stem cell transplantation is used exclusively for primary amyloidosis. High-dose melphalan followed by ASCT is currently considered the most effective therapy for AL patients. The number of the potential candidates for this combined therapy is usually limited by the strict indications in patients with clinically limited cardiac amyloidosis, representing only 5% of cases [10, 20]. This combination is thought to have a significant survival advantage over oral melphalan plus prednisone [11] with a 67% complete hematologic response and a 27% complete organ response in a series of 15 patients with an overall survival rate was 75% [10]. This applies mainly to highly-selected low-risk candidates (age <65 years, normal NT-proBNP and cardiac troponins, glomerular filtration rate > 50 mL/min), given the high treatment-related mortality, particularly in patients with heart failure and multi-organ involvement [11]. Some experts proposed criteria for candidates for stem cell transplantation. These include, age < 80 years, compensated congestive heart failure with ejection fraction > 40%, absence of pleural effusions, systolic blood pressure >90 mm Hg, and O2 saturation > 95% [4, 10]. This treatment modality results in a wide spectrum of complications ranging from pneumonia, arrhythmias, gastrointestinal bleeding, thromboembolic events, and renal failure to multiorgan failure [10].

##### The Role of Bortezomib

Bortezomib is a selective inhibitor of the 26S proteasome, a protein complex involved in the regulation of degradation of aberrant proteins as well as for the regulation of other proteins involved in the regulation of apoptosis, and cell-cycle progression. In an amyloidogenic plasma cell the overproduction of misfolded light chains causes a proteotoxic stress and other metabolic and red-ox imbalances, manifested mainly in the endoplasmic reticulum (ER). Therefore, the inhibition of the proteasome by bortezomib and blocking NF-κB activation exaggerates Enoplasmic reticulum stress and promotes apoptosis. Kastritis *et al. * [31], reported 18 cases of AL amyloidosis, among them seven patients who failed other therapies, were treated with the combination of bortezomib and dexamethasone (BD). This study showed a response rate of 94%, a hematological complete remission rate of 44% CR and organ response of 28%. The response time was o short with a median 0.9 months (range, 0.7-1.5) when compared to the 3.5 to 6 months with other effective treatments. The durability of the response to Bortezomib as well as the possible long-term side effects and toxicities were not well established, given the short follow-up of living patients (median of 11.2 months). However, a major advantage remains in the significantly shorter response time compared with those observed in other more aggressive and toxic therapies like ASCT where they can be delayed up to 36 months. Further clinical trials with long-term follow-up are needed in order to investigate and validate the use of Bortezomib in AL amyloidosis.
